# The Role of Robotics in Cardiac Surgery: Innovations, Outcomes, and Future Prospects

**DOI:** 10.7759/cureus.74884

**Published:** 2024-11-30

**Authors:** Zainoor Fida, Gul Ghutai, Zainab Jamil, Ayesha Aslam Dalvi, Muhammad Hassaan, Kainat Khalid, Umar Azam Ali, Manukrishna Sivadasan, Karishma Limbu, Nouman Anthony, Junaid H Chaudhary, Muhammad Hammad Ijaz, Sheikh Pervaiz

**Affiliations:** 1 Medicine, Khyber Teaching Hospital, Peshawar, PAK; 2 Acute Medicine/Cardiology, Wrightington, Wigan and Leigh NHS Foundation Trust, Wigan, GBR; 3 Internal Medicine, Rehman Medical Institute, Peshawar, PAK; 4 Cardiology, Rehman Medical Institute, Peshawar, PAK; 5 General Practice, Dubai Medical College for Girls, Dubai, ARE; 6 Medicine, University of Health Sciences, Lahore, PAK; 7 Internal Medicine, Ayub Medical College, Abbottabad, PAK; 8 Surgery, Jawaharlal Institute of Postgraduate Medical Education and Research, Puducherry, IND; 9 General Medicine, Nobel Medical College, Biratnagar, NPL; 10 General Medicine, Rehman Medical Institute, Peshawar, PAK; 11 Emergency Medicine, MedEast Hospital, Lahore, PAK; 12 Emergency Medicine, Allied Hospital, Faisalabad, PAK; 13 Internal Medicine, Nishtar Medical University, Multan, PAK

**Keywords:** coronary artery bypass grafting (cabg), minimally invasive surgical procedures, mitral valve repair, postoperative complications, robotic cardiac surgery, surgical robotics technology

## Abstract

In recent years, there has been a notable increase in the use of robotic technology in medical surgery, especially in heart surgery. Many advancements in surgery have been made possible by the development of these robotic devices, such as the da Vinci surgical system (Intuitive Surgical, Sunnyvale, California, United States). These advancements include improved ergonomics, three-dimensional (3D) imaging, and increased dexterity. This research evaluates the advancements, results, and potential applications of robots in heart surgery. A systematic review that adhered to the Preferred Reporting Items for Systematic Reviews and Meta-Analyses (PRISMA) principles was carried out. The PubMed and Cochrane databases underwent a thorough search that turned up articles from 2015 to 2023. Nine articles that satisfied the requirements for inclusion were evaluated for quality using the Critical Appraisal Skills Programme (CASP) checklists. Standardized templates and conventional content analysis techniques were used for data extraction and analysis, respectively. Nine studies with a range of approaches, including randomized, prospective, observational, and retrospective investigations, were included in the review. This research included a variety of robotic heart treatments, including mitral valve repair, atrial septal defect (ASD) closure, and coronary artery bypass grafting (CABG). Notable results include identical or shorter operating durations, fatality rates that are comparable to those of conventional techniques, fewer postoperative complications, and shorter hospital stays. Surgeons did, however, face an initial learning curve. Variants of the da Vinci surgical system were the most frequently used robotic systems. Robotic heart surgery has shown encouraging outcomes in terms of effectiveness, safety, and patient recovery. The dependability of robotic procedures is demonstrated by shorter operating times, reduced blood loss, a low incidence of conversion to conventional methods, and a reduction in postoperative complications. Shorter hospital stays suggest better patient outcomes and potential financial advantages. Nonetheless, the need for specific training and knowledge among surgeons is emphasized. Sustained investigation and advancement are essential for the refinement and broader use of robotic heart surgery. Robotic-assisted cardiac surgery has a promising future with a focus on improved patient outcomes, training, and procedural development.

## Introduction and background

The integration of robotic technology into medicine marks a revolutionary leap in the evolution of surgical practices [1]. Initially designed to enhance surgeons' precision and technical capabilities, robotics has now become indispensable across multiple surgical disciplines [1]. The da Vinci surgical system (Intuitive Surgical, Sunnyvale, California, United States), a pioneering example, has redefined the surgical landscape with advancements such as superior ergonomics, three-dimensional (3D) imaging, and increased dexterity [1]. These features enable surgeons to perform highly complex procedures with unparalleled accuracy and efficiency [1]. Such breakthroughs have been made possible by progress in computer processing, miniaturization, and artificial intelligence (AI) [2].

The advent of robotic surgery has brought profound and transformative changes to cardiac treatment, diverging significantly from traditional approaches [3]. Known for its intricate nature and critical precision requirements, cardiac surgery has increasingly embraced robotic innovations. Minimally invasive procedures have replaced conventional open-heart surgeries, which were previously associated with substantial trauma and extended recovery times [4]. This shift has been further propelled by robotic technology [3]. Today, robotic cardiac surgery encompasses a broad range of interventions, including coronary artery bypass grafting (CABG) and valve repairs [5]. Renowned for its exceptional accuracy, reduced patient trauma, and accelerated recovery periods, robotic-assisted surgery is rapidly becoming the preferred choice in modern cardiac care [6]. The adoption of robotic systems has significantly improved surgical outcomes while broadening the scope of possibilities in cardiac procedures [7]. The ability of robotic systems to execute precise movements within confined spaces, such as the chest cavity, has been instrumental in addressing the challenges posed by complex cardiac surgeries [8]. Given the delicate nature of cardiac tissues and the need for meticulous intervention, this technological advancement holds exceptional significance [8].

The field of robotic cardiac surgery is continually evolving as researchers and clinicians explore and implement novel techniques. Applications of robotic systems have expanded beyond mitral valve surgery and coronary revascularization, addressing diverse and complex cardiac conditions [8]. Accumulating empirical evidence supports the effectiveness and safety of robotic cardiac surgery, reinforcing its potential to become a standard practice in the field [3]. However, this progress is not without challenges. Key obstacles include issues with cardiopulmonary bypass (CPB) management, myocardial protection during robotic procedures, and the requirement for specialized training [3,8]. Additionally, the financial burden of implementing and maintaining robotic systems poses significant challenges, particularly for healthcare systems in middle-income countries like Colombia [4]. Despite these barriers, the trajectory of robotic cardiac surgery remains optimistic, driven by technological advances and increasing global acceptance. This innovative approach has the potential to revolutionize cardiac care by enhancing safety, reducing invasiveness, and improving patient outcomes.

While the short-term benefits of robotic cardiac surgery, such as reduced hospital stays and quicker recoveries, are well-documented [9], there remains a lack of extensive longitudinal research on its long-term outcomes and cost-effectiveness. Critical areas like mortality rates, quality of life, and cost-efficiency over time remain underexplored, particularly as robotic technology continues to evolve [10]. As new equipment and procedures are introduced, consistent evaluation of their long-term efficacy and impact is vital [10]. Addressing this research gap is crucial for providing comprehensive insights into the value of robotic cardiac surgery. Such knowledge would benefit not only patients and healthcare providers but also policymakers tasked with resource allocation and technology investment [11]. By examining these aspects, this study aims to support informed decision-making, ensuring that medical technology investments yield tangible and sustainable benefits for patients and healthcare systems [11].

This research is motivated by the need to bridge the gap between technological advancements and their clinical integration. While robotics in cardiac surgery has made significant technological strides, clinical evidence supporting its widespread adoption remains limited. The primary goal of this study is to generate empirical data to inform clinical practices and policy frameworks. Additionally, with the healthcare industry increasingly prioritizing value-based care, understanding the cost-effectiveness of robotic surgery is imperative. By assessing whether its higher initial costs are offset by improved long-term outcomes and healthcare delivery efficiency, this study seeks to provide meaningful contributions to the dynamic field of cardiac surgery [12].

The objectives of this study include a comprehensive synthesis of existing data and advancements in robotic cardiac surgery. This analysis focuses on cutting-edge developments, clinical outcomes, and areas for future exploration. Specifically, the study evaluates the immediate- and long-term impacts of robotic technology on cardiac surgical procedures, identifies the key technological drivers behind this progress, and examines the implications for surgical techniques and patient care. It also highlights the challenges and limitations associated with robotic cardiac surgery, such as financial, technological, and training-related issues.

## Review

Materials and methods

Review Design and Search Strategy

The systematic review was meticulously conducted in strict adherence to the criteria outlined by the Preferred Reporting Items for Systematic Reviews and Meta-Analyses (PRISMA). The protocol was collaboratively developed and unanimously approved by all authors involved. The primary objective of this study was to comprehensively examine the role of robots in cardiac surgery, with an emphasis on the latest achievements, outcomes, and potential future developments in the field. The search strategy was meticulously crafted to capture a wide range of relevant scientific literature. A comprehensive search was conducted on PubMed and Cochrane databases, covering the period from 2015 to December 2022, with the final search extending into 2023. Two independent researchers, A and B, conducted the investigation using a combination of Medical Subject Headings (MeSH) terms, phrases, and free-text terms. Primary search terms included "robotic cardiac surgery", "robot-assisted cardiac procedures", and "minimally invasive cardiac surgery". Boolean operators (OR and AND) were employed to combine these terms. Additionally, truncation was strategically used to enhance the retrieval of relevant scholarly resources. Besides electronic searches, a manual search of references in influential articles and review papers was conducted to identify any missed studies.

Quality Assessment

The selected articles' quality was evaluated using the checklists provided by the Critical Appraisal Skills Programme (CASP). These checklists were employed to assess several study designs, including randomized controlled trials, observational studies, and qualitative research. The initial evaluation was carried out independently by three authors, who used a scoring system where a score of 2 indicated full compliance, 1 indicated partial compliance, and 0 indicated non-compliance or inapplicability. After completing their individual assessments, the authors convened to compare evaluations and engage in discussions. This collaborative approach ensured a thorough and unbiased assessment of each study. Following this rigorous assessment, 475 papers were excluded due to low-quality ratings, leaving nine studies that met the moderate- or high-quality criteria.

Data Extraction and Categorization

Data extraction followed a standardized template, collecting crucial information such as the primary author's name, publication year, study country, research design, specific robotic technology used, study location, participant characteristics, and significant outcomes. The extracted data were coded and classified into subcategories. Through an analysis of the relationships among these codes, broader categories were formed. A series of iterative discussions among the authors determined the final categories and subcategories. The goal was to reach a consensus on the core topics and findings of the review.

Results

Study Selection

**Figure 1:**
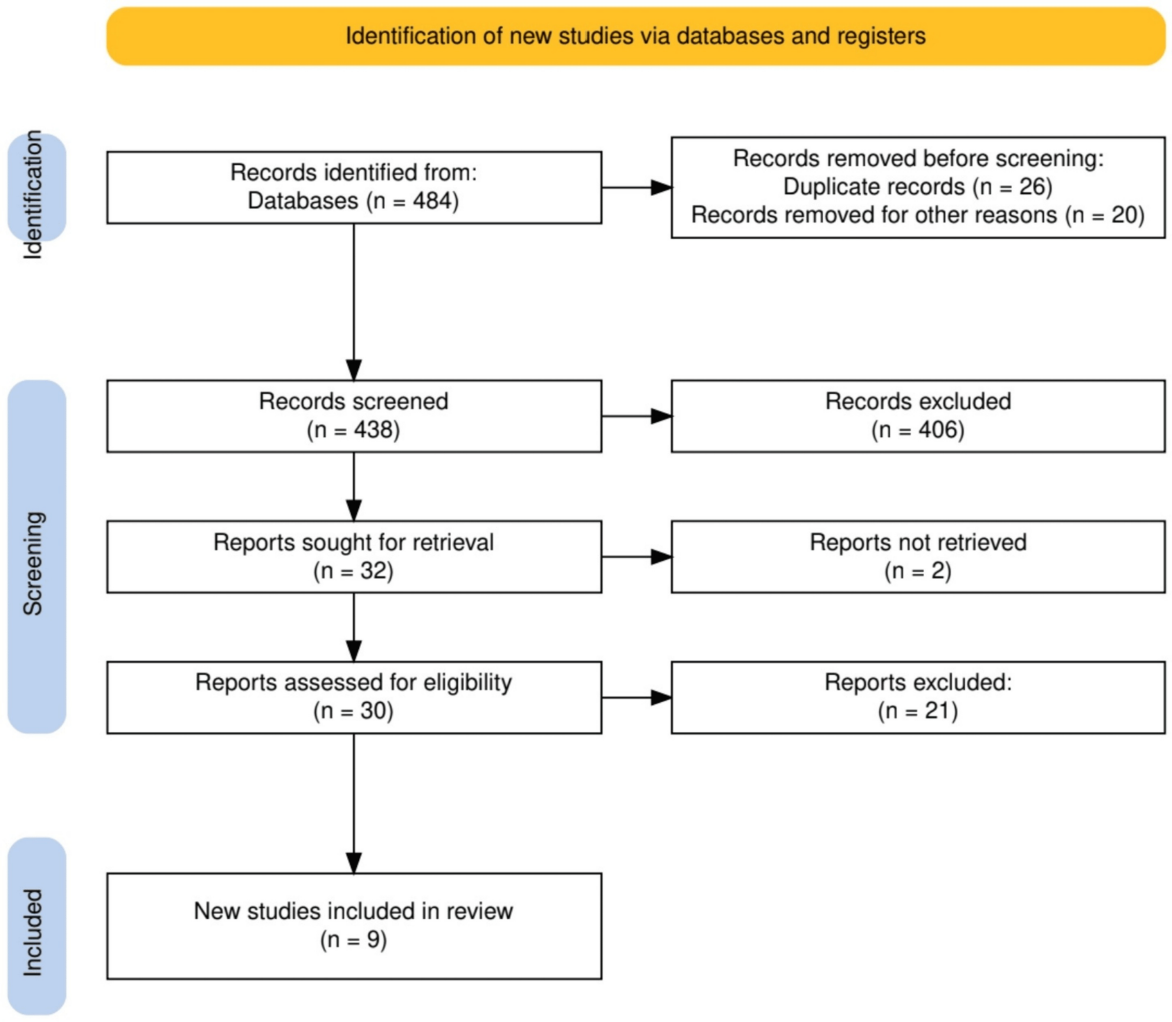
The PRISMA flowchart representing the study selection process. PRISMA: Preferred Reporting Items for Systematic Reviews and Meta-Analyses

The initial database search retrieved 484 records. Out of which, 26 were excluded on the basis of duplicate, while 20 were excluded due to the review article. The remaining 438 articles were screened for their title/abstract, and 32 publications were deemed for full-text screening. Out of which, two publications were excluded as their full text was not accessible. The full-text screening of the remaining 30 articles retrieved nine articles to be included in the interview. The detailed process of screening and the reason for exclusion are illustrated in the PRISMA diagram, in Figure 1.

Study Characteristics

This review includes nine studies of various types, including one randomized [13], two prospective [14,15], one observational [16], and five retrospective studies [17,18]. These studies were conducted in different regions, including London [13], the USA [16], Japan [14,15], Finland [17], Turkey [18], Korea [19], and the Netherlands [20,21]. Depending on the study, overall sample sizes (n=1103) range from eight to 605 patients, including a mix of male and female participants. The mean ages of the participants, as reported, exhibit considerable variation, stretching from 43.4 to 69 years. Two investigations reported follow-up data [17,20]. Table 1 summarizes the characteristics of the nine studies on robotic-assisted surgical procedures in cardiac surgery.

**Table 1:** Study characteristics of the included papers. UK: United Kingdom; USA: United States of America; CABG: coronary artery bypass grafting; ASD: atrial septal defect; RMVP: robotic mitral valve repair; NR: not reported; AF: atrial fibrillation

Reference	Detailed study type	Region	N	Sex	Age, y∗	Cardiac surgery	Surgical system
Arujuna et al., 2015 [13]	Randomized prospective study	London, UK	40	M=115	59 (10.8)	Left atrial ablation	NavXTM (St. Jude Medical Inc., Saint Paul, Minnesota, United States) or CARTO XP (Biosense Webster Inc., Irvine, California, United States)
Giambruno et al., 2018 [16]	Observational study	USA	605	F=75	52.9 (15.1)	CABG	Automated Endoscopic System for Optimal Positioning (AESOP), Zeus telemanipulation system, da Vinci Si surgical system (Sunnyvale, California, United States)
Ishikawa et al., 2018 [14]	Prospective study	Japan	8	M=27	43.4±14.6	ASD	da Vinci surgical system
Kesävuori et al., 2018 [17]	Retrospective study	Finland	142	M=28	54+13.8	RMVP	da Vinci Si surgical system
Senay et al., 2021 [18]	Retrospective study	Turkey	123	F=150; M=455	61.2±10.7	RMVP	da Vinci Xi and Si surgical system
Spanjersberg et al., 2022 [21]	Retrospective study	Netherlands	107	M=2	NR	CABG	da Vinci Si surgical system
van der Heijden et al., 2022 [20]	Retrospective study	Netherlands	23	F=75 (60.9)	65.3 (8.6)	AF ablation CABG	da Vinci Si surgical system
Yahagi et al., 2020 [15]	Prospective study	Japan	10	F=5 (22)	69 (8)	RMVP	NR
Yun et al., 2022 [19]	Retrospective study	Korea	45	NR	NR	ASD	da Vinci Si surgical system

Surgical Type and Surgical System

Surgical procedures performed in different studies included CABG [21], left atrial ablation [13,20], atrial septal defect (ASD) closure [14,19], and robotic mitral valve repair (RMVP) [17,18]. Various surgical systems were utilized in each of the investigations. Arujuna et al. [13] utilized NavXTM (St. Jude Medical Inc., Saint Paul, Minnesota, United States) and CARTO XP (Biosense Webster Inc., Irvine, California, United States), while Giambruno et al. [16] employed Automated Endoscopic System for Optimal Positioning (AESOP) and the Zeus telemanipulation system (Sunnyvale, California, United States). da Vinci surgical systems were implemented in seven studies, with the exception of one study which did not report any surgical system [16,14,17,18,21,20].

Atrial fibrillation (AF) was documented in two studies, Arujuna et al. [13] and van der Heijden et al. [20], with reported prevalence rates of 33.1% and 2.8%, respectively. Left atrial (LA) diameter varied across studies, with Spanjersberg et al. [21] reporting a mean diameter of 15.4±4.6 mm. Ejection fraction (EF) data were available in five studies, with the majority reporting values above 50%. Notably, Kesävuori et al. [17] reported a mean LA diameter of 3.4±3.5 cm, while Yun et al. [19] did not provide specific information on AF, LA diameter, or EF. The values represented in different studies are detailed in Table 2.

**Table 2:** Atrial fibrillation, LA diameter, and ejection fraction as reported in various studies. LA: left atrial; NR: not reported

Reference	Atrial fibrillation	LA diameter (mm)	Ejection fraction
Kesävuori et al., 2018 [17]	47 (33.1)	49.9 (7.1)	>50%=129; ≤50%=13
Senay et al., 2021 [18]	29	NR	≤50%=5
Yun et al., 2022 [19]	7 (15.6)	NR	NR
Arujuna et al., 2015 [13]	NR	(cm) 3.4+3.5	NR
Giambruno et al., 2018 [16]	NR	NR	NR
Ishikawa et al., 2018 [14]	NR	15.4±4.6	70.1±8.4%
Spanjersberg et al., 2022 [21]	3 (2.8)	NR	≤50%=25
van der Heijden et al., 2022 [20]	23	39 (5)	57 (7)
Yahagi et al., 2020 [15]	NR	NR	NR

Comorbidities

The patients who are undergoing robotic-assisted cardiac surgery exhibit a diverse range of comorbidities, with varying frequencies reported across the studies outlined in Table 3. The patient presents with a range of comorbidities, namely, diabetes (n=7), hypertension (n=6), chronic lung disease (n=6), peripheral vascular disease (n=1), coronary artery disease (n=1), thyroid disorder (n=1), smoking history (n=1), myocardial infarction (n=5), stroke (n=3), transient ischemic attack (n=2), percutaneous coronary intervention (n=1), and previous cardiac surgery (n=1).

**Table 3:** Comorbidities represented by patients. NR: not reported; N (%): number (percentage)

Comorbidities	Kesävuori et al., 2018 [17]	Senay et al., 2021 [18]	Yun et al., 2022 [19]	Arujuna et al., 2015 [13]	Giambruno et al., 2018 [16]	Ishikawa et al., 2018 [14]	Spanjersberg et al., 2022 [21]	van der Heijden et al., 2022 [20]	Yahagi et al., 2020 [15]
Diabetes, N (%)	6 (4.2)	18 (14.6)	1 (2.2)	1 (2.5)	515 (85.12)	NR	20	4 (17)	NR
Hypertension, N (%)	48 (33.8)	40 (32.52)	5 (11.1)	11 (27.5)	NR	NR	56 (52.3)	22 (96)	NR
Chronic lung disease, N (%)	7 (4.9)	1 (0.8)	1 (2.2)	NR	38 (6.2)	NR	15 (14)	4 (17)	NR
Peripheral vascular disease, N (%)	NR	NR	NR	NR	21 (3.47)	NR	NR	NR	NR
Coronary artery disease, N (%)	NR	NR	NR	1 (2.5)	NR	NR	NR	NR	NR
Thyroid disease, N (%)	NR	NR	NR	2 (5)	NR	NR	NR	NR	NR
Smoking, N (%)	NR	NR	NR	6 (15)	NR	NR	NR	NR	NR
Myocardial infarction, N (%)	1 (0.7)	5 (4.06)	NR	NR	69 (11.4)	NR	20 (18.7)	5 (22)	NR
Stroke, N (%)	1 (0.7)	NR	NR	NR	19 (3.1)	NR	NR	2 (9)	NR
Transient ischemic attack, N (%)	NR	NR	NR	NR	9 (1.48)	NR	NR	1 (4)	NR
Percutaneous coronary intervention, N (%)	0	NR	NR	NR	NR	NR	11 (5.7)	2 (9)	NR
Previous cardiac surgery, N (%)	0	7	NR	NR	NR	NR	NR	NR	NR

Operative Time

A majority of studies (n=7) reported a comparable or reduced operative time in robotic-assisted cardiac surgeries compared to traditional approaches [13-15,17,18,20]. CPB time was measured in five studies [14,17,18,15,19]. In all studies, the robotic platform demonstrated efficiency in complex procedures such as mitral valve repair and CABG. Evidence suggests a trend towards reduced blood loss in robotic surgeries, contributing to improved postoperative recovery [13,14,17,18,21,20,15,19].

Mortality Rates

The overall mortality rates were similar between robotic and traditional cardiac surgery groups, indicating the safety of robotic interventions. Interestingly, a few studies reported a zero mortality rate [14,21,20].

Complications and Conversion

Fewer postoperative complications, including myocardial infarction, AF, and wound complications, were observed in the robotic-assisted surgeries. Little conversion was reported in three studies, while three studies reported zero conversion. Reoperation due to bleeding was also reported in two studies in a limited number of patients.

Hospital Stay

Robotic surgeries were associated with a shorter hospital stay compared to conventional approaches, suggesting potential economic benefits [13,14,17,18,21,20,15,19]. A lower number of readmissions was reported in only two studies.

Learning Curve

Only one study acknowledged an initial learning curve for surgeons adopting robotic techniques, with proficiency gained over time [17].

Quality Assessment

The quality assessment of the articles was conducted using the CASP checklist. While all the studies addressed a clearly focused issue (Q1), only one study was a randomized controlled trial [13]. This study met the criteria for Q2 and Q3, while most other studies were either unclear (NC) or did not meet the criteria. Several studies [16,14] did not provide sufficient data regarding group assignment or blinding, resulting in lower scores for Q3 and Q4. Two studies [17,18] provided comprehensive data on important clinical outcomes, while others reported relevant but less comprehensive outcomes. Overall, the quality of the papers was mixed, with some studies meeting most criteria and others being unclear in certain areas, but the general assessment indicates the papers were of good quality. A summary has been provided in Table 4.

**Table 4:** Quality assessment of articles according to the CASP checklist. Y: yes (indicates that the criterion was met); N: no (indicates that the criterion was not met); NC: not clear; CASP: Critical Appraisal Skills Programme

	Arujuna et al., 2015 [13]	Giambruno et al., 2018 [16]	Ishikawa et al., 2018 [14]	Kesävuori et al., 2018 [17]	Senay et al., 2021 [18]	Spanjersberg et al., 2022 [21]	van der Heijden et al., 2022 [20]	Yahagi et al., 2020 [15]	Yun et al., 2022 [19]
Did the study address a clearly focused issue?	Y	Y	Y	Y	Y	Y	Y	Y	Y
Was the assignment of patients to treatments randomized?	Y	N	N	N	N	N	N	N	N
Were patients, health workers, and study personnel blinded?	NC	N	N	N	N	N	N	N	N
Were the groups similar at the start of the study?	Y	NC	NC	Y	N	Y	N	Y	N
Aside from the experimental intervention, were the groups treated equally?	NC	NC	NC	NC	NC	NC	NC	NC	NC
Were all of the patients who entered the study properly accounted for at its conclusion?	Y	Y	Y	Y	Y	Y	Y	Y	Y
How large was the treatment effect?	Y	N	Y	Y	Y	Y	NC	Y	NC
How precise was the estimate of the treatment effect?	Y	Y	Y	Y	Y	Y	Y	Y	Y
Can the results be applied in your context? (or to the local population?)	Y	N	Y	Y	Y	Y	Y	Y	Y
Were all clinically important outcomes considered?	NC	NC	NC	Y	Y	NC	NC	NC	NC
Are the benefits worth the harms and costs?	Y	Y	Y	Y	Y	Y	Y	Y	Y

Discussion

Robotic surgery has consistently demonstrated promising outcomes across various cardiac procedures, including valve repair, CABG, and ASD repair. These advancements highlight the growing acceptance and effectiveness of robotic technology in complex cardiac interventions. The successful implementation of two-port robotic cardiac surgery for ASD treatment, as reported by Yun et al., underscores the potential of minimally invasive techniques to achieve clinical goals with minimal patient trauma and quicker recovery times [19]. Similarly, robotic mitral valve surgery has proven to be both safe and efficacious, particularly in patients with comorbidities like obesity, where traditional sternotomy might pose higher risks. This finding emphasizes that obesity should not be a contraindication for robotic procedures [18]. Remarkably, the rate of valve repair in robotic groups was observed to be 98.6%, comparable to 97.9% in sternotomy groups, as reported by Kesävuori et al., further validating the reliability of robotic approaches [17]. Moreover, the use of transesophageal echocardiography (TEE) has enhanced procedural precision by enabling the accurate selection of annuloplasty ring sizes and artificial chordae lengths, especially in conditions like Barlow's disease [15].

In the realm of ablation techniques, robotic systems have shown the potential to improve long-term outcomes. For instance, robotic ablation has been associated with increased late gadolinium enhancement, potentially reducing the likelihood of future reoperations [13]. Another notable advancement is the integration of unilateral left-sided thoracoscopic AF ablation with minimally invasive direct coronary artery bypass (MIDCAB). This technique, specifically involving left internal mammary artery to left anterior descending artery (LIMA-LAD) grafting, has been shown to be both feasible and reliable. It offers a tailored solution for patients with concurrent AF and significant coronary artery narrowing, highlighting the versatility of robotic systems in addressing multifaceted cardiac conditions [20].

Robot-assisted CABG has emerged as a compelling alternative to traditional CABG, combining safety, feasibility, and efficacy. According to Giambruno et al., robotic CABG procedures not only match the clinical outcomes of conventional approaches but also bring additional advantages such as reduced blood loss, shorter hospital stays, and faster recovery times [16]. Furthermore, Spanjersberg et al. reported that robotic CABG significantly increased the likelihood of early discharge to home compared to off-pump CABG, underscoring its potential for improving patient throughput and reducing healthcare costs [21].

Despite these advancements, robotic cardiac surgery is not without challenges. One of the main hurdles is the steep learning curve associated with adopting robotic systems. Proficiency in robotic techniques requires significant training and experience, which can initially limit the widespread adoption of these technologies. Additionally, the high upfront costs of robotic systems, such as the da Vinci surgical system, and their maintenance present financial barriers, particularly for healthcare systems in resource-limited settings. Addressing these challenges will require a multifaceted approach, including enhanced surgeon training programs, financial incentives for hospitals, and further technological innovations to make robotic systems more affordable.

Furthermore, while the current evidence highlights excellent short-term outcomes, there remains a paucity of data on the long-term benefits of robotic cardiac surgery. Future studies should focus on long-term patient outcomes, such as survival rates, quality of life, and cost-effectiveness, to provide a more comprehensive understanding of its value in clinical practice. The continued refinement of robotic technology, combined with efforts to address its financial and educational barriers, has the potential to establish robotic-assisted surgery as a gold standard for various cardiac procedures.

Robotic cardiac surgery is a transformative innovation with proven safety and efficacy across a range of procedures. It offers a minimally invasive alternative with significant benefits for patient recovery and hospital efficiency. As the field continues to evolve, sustained research and development will be critical to overcoming existing limitations and ensuring the broader adoption of robotic technologies in cardiac care.

## Conclusions

The utilization of robots in cardiac surgery has shown encouraging results concerning effectiveness, security, and recuperation time for patients. The efficacy of robotic systems is shown by the shorter operating durations and blood loss, as well as comparable or lower death rates when compared to conventional procedures. The low rate of conversions to conventional techniques and postoperative problems is another evidence of these technologies' dependability. Shorter hospital stays are indicative of both better patient outcomes and possible financial savings for healthcare organizations. However, the research also highlights the significance of the learning curve related to robotic surgery, highlighting the need for specific education and expertise. Based on the overall quality of evaluated papers, robotic cardiac surgery is a promising subject that requires ongoing study and monitoring to be optimized. Robotic-assisted cardiac surgery seems to have a promising future with an emphasis on method refinement, improved training for surgeons, and improved patient outcomes.
